# Mendelian randomization reveals apolipoprotein B shortens healthspan and possibly increases risk for Alzheimer’s disease

**DOI:** 10.1038/s42003-024-05887-2

**Published:** 2024-02-24

**Authors:** Leah Martin, Brian B. Boutwell, Carmen Messerlian, Charleen D. Adams

**Affiliations:** 1 Department of Epidemiology, Harvard T. H. Chan School of Public Health, Boston, MA USA; 2 School of Applied Sciences, University of Mississippi, University, Jackson, MS USA; 3 John D. Bower School of Population Health, University of Mississippi Medical Center, Jackson, MS USA; 4 Department of Obstetrics and Gynecology, Massachusetts General Hospital Fertility Center, Boston, MA USA; 5 Department of Environmental Health, Harvard T. H. Chan School of Public Heath, Boston, MA USA

**Keywords:** Heritable quantitative trait, Alzheimer's disease

## Abstract

Apolipoprotein B-100 (APOB) is a component of fat- and cholesterol-transporting molecules in the bloodstream. It is the main lipoprotein in low-density lipoprotein cholesterol (LDL) and has been implicated in conditions that end healthspan (the interval between birth and onset of chronic disease). However, APOB’s direct relationship with healthspan remains uncertain. With Mendelian randomization, we show that higher levels of APOB and LDL shorten healthspan in humans. Multivariable Mendelian randomization of APOB and LDL on healthspan suggests that the predominant trait accounting for the relationship is APOB. In addition, we provide preliminary evidence that APOB increases risk for Alzheimer’s disease, a condition that ends healthspan. If these relationships are causal, they suggest that interventions to improve healthspan in aging populations could include strategies targeting APOB. Ultimately, given that more than 44 million people currently suffer from Alzheimer’s disease worldwide, such interventions are needed.

## Abstract (web summary)

A Mendelian randomization study suggests that apolipoprotein B potentially contributes to shortened healthspan and increased risk for Alzheimer’s disease.

## Introduction

Apolipoprotein B-100 (APOB) is a component of fat- and cholesterol-transporting molecules in the bloodstream: namely, it is a building block of very low-density lipoproteins (VLDLs), intermediate-density lipoproteins (IDLs), and low-density lipoproteins (LDLs). Elevated levels of circulating APOB^1^ and APOB-containing lipoproteins^2^ are strongly associated with lifespan (the interval between birth and death). Likewise, APOB has been implicated in conditions that end healthspan (the interval between birth and onset of chronic disease)^3–5^. A condition that ends healthspan is Alzheimer’s disease (AD), the most common cause of dementia^6^. More than 44 million people (about twice the population of the state of New York) suffer from AD worldwide. The prevalence of AD is likely underestimated, however, since AD may begin decades before memory loss is noticed by a person losing memory and thus go undiagnosed for years^7^. Moreover, as the populations ages (as lifespans increase), more people are living with AD in their later years. For these reasons and because APOB is a potentially environmentally modifiable factor, we take a particular interest in circulating APOB in relationship to healthspan and AD. If the relationships between APOB, AD, and healthspan are causal, this suggests that efforts to modify levels of APOB are a potential avenue for protecting cognitive function as the population ages.

To that end, although evidence exists to support the role of APOB in other diseases that end healthspan, such as heart disease and stroke^3–5^, whether APOB directly ends healthspan and causes AD is uncertain. Support for the role of APOB in causing AD comes from a growing body research in humans and animal models. Wingo et al. ^8^ observed that rare *APOB*-coding variants were more abundant than expected in early-onset AD (EOAD) cases after adjusting for the apolipoprotein E ε4 (*APOE E4*) allele^8^. (Mutations in *APOE* are the most common genetic risk factor for AD^9^ and are what probably come to mind when thinking of apolipoproteins and AD). The finding by Wingo and colleagues comports with earlier observations. Caramelli et al. ^10^ and Kuo et al. ^11^ found higher serum concentrations of APOB in AD patients than elderly controls^10,11^. This led Caramelli and colleagues to suggest that APOE may not be the only factor influencing AD pathogenesis^10^. Likewise, Namba, Tsuchiya, and Ikeda (1992) detected APOB immunoreactivity in senile plaques, vascular amyloids, and neurofibrillary tangles (NFT) in the brains of two patients with AD^12^. They suggested that APOB may be involved in the formation of NFT and amyloid^12^.

Picard et al. ^13^ recently detected APOB in the cerebral spinal fluid (CSF) of subjects classified as at risk for AD. They concluded that its presence in the CSF may represent an early biomarker of tau pathology in AD^13^. Similar results, though, have been observed in the CSF of patients with cerebrotendinous xanthomatosis, consistent with the view that APOB detected in CSF is the result of disease-induced weakening of the blood-brain barrier, BBB (the interface between circulating blood and neural tissue)^12–14^.

Damage to both the structure and functions of the BBB have previously been reported in AD patients^15^. Moreover, Bowen, Kaye, and Quinn (2012) reported dyslipidemia in 47% of the mild-to-moderate cases of AD they studied and in 75% of AD cases with BBB impairment^16^. The latter suggests that dyslipidemia specifically may impair the BBB. It is worth noting, though, that ref. ^13^ found no evidence that presence of APOB in CSF resulted from BBB leakage of circulating APOB in their sample, a result consistent with their suggestion that localized production of APOB in the brain is possible. If evidence emerges suggesting causal effects of APOB, this point is worth revisiting when contemplating mechanisms and causal pathways.

Additional support for the role of APOB in AD comes from APOB-100 transgenic mice models. Löffler et al. ^17^ suggest that APOB is a vasculature risk factor for AD that may affect brain aging and cognitive function. They found that overexpression of *APOB* in human APOB-100 transgenic mice caused memory decline^17^. Others from the same lab observed high plasma levels of triglycerides, cognitive impairment, and increased BBB permeability in the hippocampuses of the transgenic mice^15^. While the APOB bound to VLDL, IDL, and LDL is not believed to be produced in the human brain^18^, *APOB* mRNA has been observed in the brains of the APOB-100 transgenic mice^19^. Together with the human data mentioned above, this reinforces the possibility that APOB could be involved in the pathogenesis of AD, and thus, contribute to brain-based ending of healthspan. Yet causality is uncertain.

When it comes to causality, Mendelian randomization (MR) is an approach that can overcome some of the gaps in causal inference when a randomized controlled trial (RCT) in humans has not been done. Here, we used MR, a genetic causal inference technique, to interrogate links between (a) circulating metabolites and healthspan and (b) between APOB and low-density lipoprotein cholesterol (LDL) and AD. MR uses effect estimates from genetic variants strongly associated with exposure traits (i.e., independent variables) in models instead of the traits themselves. Due to the random transmission of alleles from parent to offspring, using genetic variants strongly associated with traits (instead of the traits) quasi-randomizes subjects on characteristics other than the trait acting as the intervention (i.e., the exposure). Thus, MR mimics an RCT. Crucially, because of this, MR avoids most sources of non-genetic confounding that can distort causal estimates in observational designs^20^.

In this MR study, we show that higher levels of APOB and LDL shorten healthspan in humans and, with multivariable MR, observe that the predominant trait accounting for the relationship is APOB. In addition, we provide preliminary evidence that APOB increases risk for AD. If these relationships are causal, they suggest that interventions to improve healthspan and reduce cognitive decline in aging populations could include strategies targeting APOB.

## Results

### Study overview

**Fig. 1:**
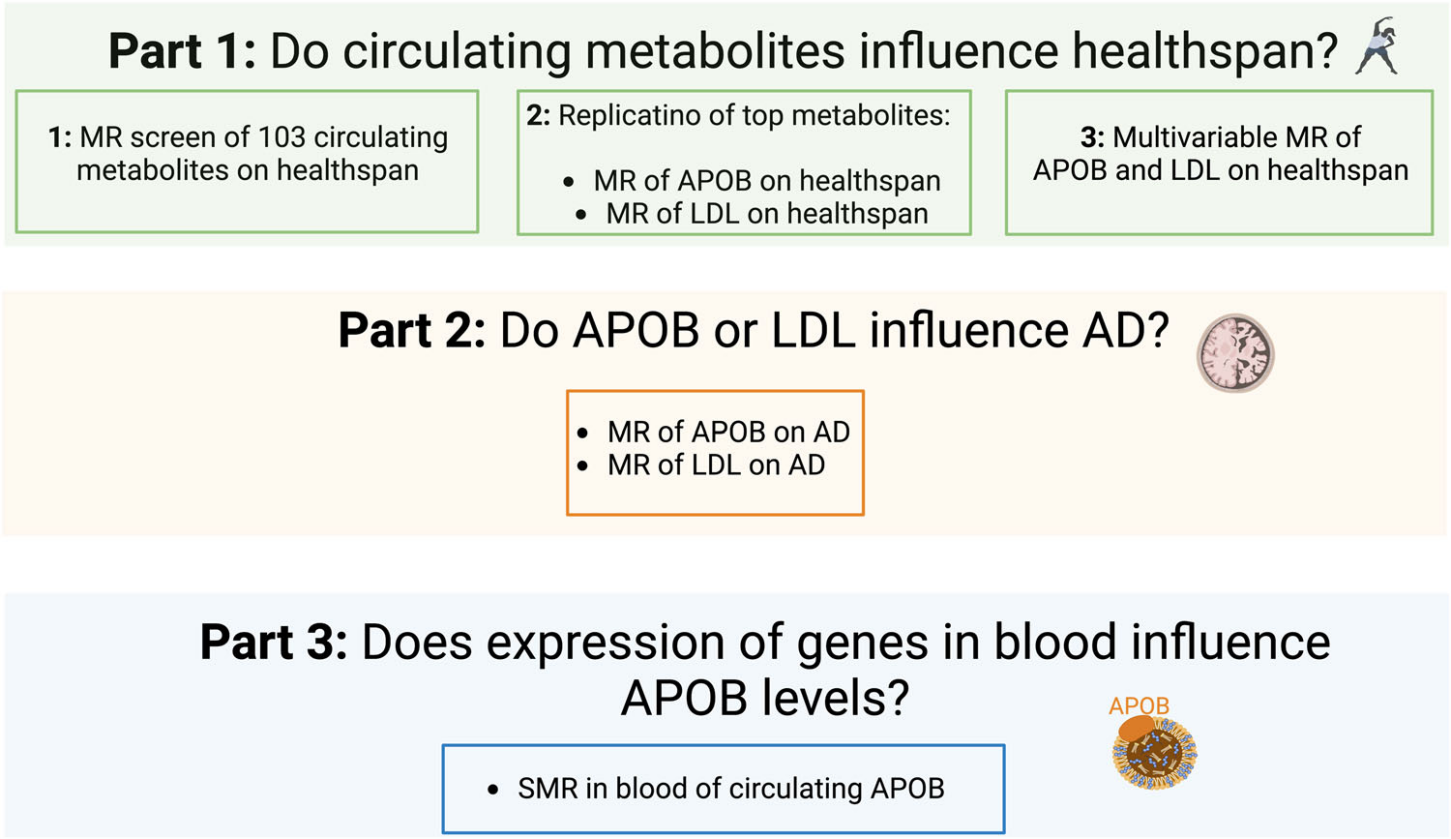
Study overview. The study overview presents our scientific questions and the MR designs we employed to investigate them. Part 1 (green) has three components: (1) an MR screen of circulating metabolites on healthspan; (2) a replication of top findings from the metabolites screen (i.e., of apolipoprotein B [APOB] and low-density lipoprotein cholesterol [LDL]); and (3) a multivariable MR analysis including both APOB and LDL as exposures on healthspan. Part 2 (orange) consists of an MR appraisal of whether APOB or LDL influence risk for Alzheimer’s disease (AD). Part 3 (blue) comprises a transcriptomic summary-data based MR (SMR) analysis to identify genes whose expression in blood influence circulating levels of APOB.

**Fig. 2:**
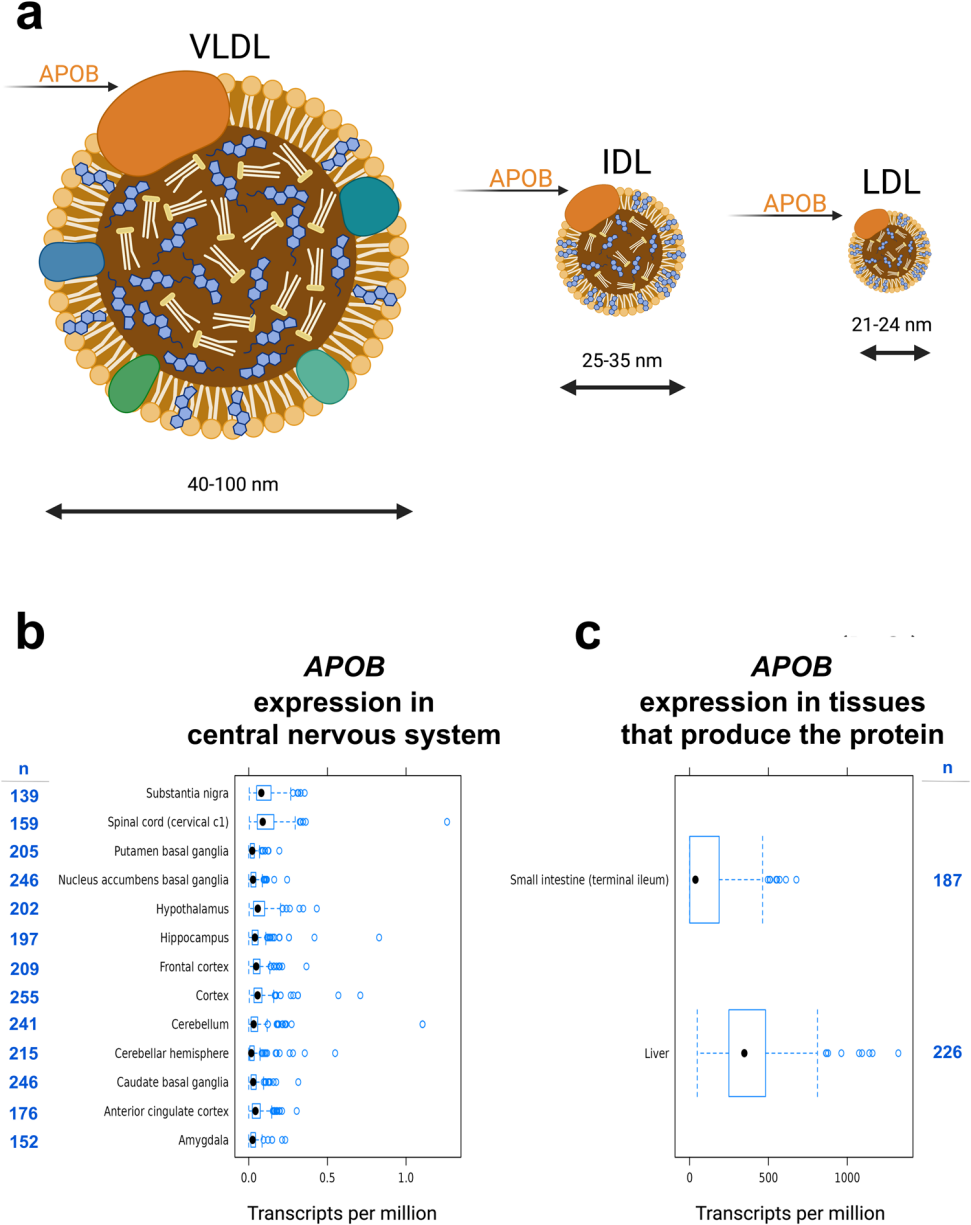
Major classes of apolipoprotein B (APOB)-containing lipoproteins and *APOB* gene expression in various brain regions and in tissues that produce the protein (small intestine and liver). **a** Cartoon of the major APOB-containing lipoproteins (excluding chylomicrons). APOB is the main lipoprotein in very-low density lipoprotein (VLDL), intermediate density lipoprotein (IDL), and low-density lipoprotein (LDL)^60^. ^55^Box plots showing the median values (black dots), interquartile ranges (blue squares, where the left edge represents the lower quartile and the right edge the upper quartile), and outliers (blue circles) of *APOB* gene expression (units = transcripts per million) in (**b**) brain and spinal cord and (**c**) the main tissues that produce the circulating APOB protein (small intestine and liver).

To implement MR, we integrated summary statistics (Table 1) from various genome-wide association (GWA) studies. Figure 1 contains an overview of our approach. First, we started by integrating the GWA studies for circulating metabolites (using metabolite quantitative trait loci, metQTLs)^4,21^ and healthspan^22^. With these integrated data, we performed an MR screen of 103 circulating nuclear-magnetic resonance (NRM) metabolites (sample sizes up to 24,925) and examined them in relation to healthspan (sample size = 300,447). This metabolite screen revealed that APOB and lipids containing LDL shorten healthspan. Next, we replicated these findings using larger GWA studies of APOB (sample size = 439,214) and LDL (sample size = 440,546) from the UK Biobank (UKBB) and performed a multivariable MR analysis to assess the direct effects of APOB and LDL on healthspan, given that APOB is a component of LDL (see Fig. 2) The multivariable MR analysis indicated that APOB has a direct effect on healthspan when accounting for LDL. Having observed that APOB shortens healthspan and has a direct effect on healthspan when accounting for LDL, we sought to determine whether it increases risk for AD. As part of our preliminary investigation into this, because circulating APOB-containing lipids are prevented from crossing the blood-brain barrier under normal circumstances, we wondered whether *APOB* was expressed in brain. Circulating APOB is primarily produced in liver and small intestine, but we observed that it is expressed in very small amounts in brain and spinal cord tissue. We observed this by examining gene expression in 13 central-nervous system tissues (12 brain regions and spinal cord) and in liver and small intestine from participants in the Gene-Tissue Expression (GTEx) project. See Fig. 2 and Supplementary Data 1. Next, we integrated summary statistics for (UKBB) APOB and (UKBB) LDL with an AD GWA study (21,982 cases; 41,944 cognitively normal controls) and ran MR on each. MR revealed that APOB, but not LDL, increases risk for AD. Having observed that APOB shortens healthspan and increases risk for AD, we performed transcriptomic summary-data based MR (SMR) to identify candidate gene targets whose expression in blood influences the levels of circulating APOB.

**Table 1:** Data sources

Trait	GWA study data source: consortium and website for obtaining the data	Descriptive notes	Sample size
Healthspan	Zenin et al.^22^; UK Biobank (UKBB)^53,54^; https://www.gwasarchive.org/^55^	Living free from congestive heart failure (CHF), myocardial infarction (MI), chronic obstructive pulmonary disease (COPD), stroke, dementia, diabetes, cancer, and death; mid-life participants aged 37 to 73	300,447
Lifespan	LifeGen study by Timmers et al.^41^; UKBB^53,54^; https://datashare.ed.ac.uk/handle/10283/3209/^56^	Parental lifespans; participants aged 40 to 107	up to 640,189
Late-onset Alzheimer’s disease (AD)	Kunkle et al.^42^; MRC IEU ID: ieu-b-2^44,57^; https://pubmed.ncbi.nlm.nih.gov/30820047/	Subjects from: International Genomics of Alzheimer’s Project (IGAP). Mean age at onset of 76 for cases, and mean age at examination of 71 for controls	21,982 cases; 41,944 cognitively normal controls
AD and AD-by-proxy	Jansen et al.^27^; https://www.ebi.ac.uk/gwas/publications/30617256^58^	Subjects from four consortia: Alzheimer’s disease working group of the Psychiatric Genomics Consortium, (PGC-ALZ), (IGAP), and the Alzheimer’s Disease Sequencing Project (ADSP), UKBB.	71,880 clinically-diagnosed and AD-by-proxy cases and 383,378 controls.
103 Circulating Metabolites	Kettunen et al.^21^; MRC IEU ID: met-c^44,57^; https://pubmed.ncbi.nlm.nih.gov/27005778/	NMR-measures for diverse metabolic pathways: amino acids, fatty acids, lipids, lipoproteins, and small molecules; mean age of 46 for participants	up to 24,925
Apolipoprotein B (APOB)	Richardson et al.^4^/UKBB^53,54^; MRC IEU ID: ieu-b-108^44,57^; https://gwas.mrcieu.ac.uk/datasets/ieu-b-108/	Circulating, non-fasted measure of APOB; mean age of 57 for participants	439,214
Low-density lipoprotein cholesterol (LDL)	Richardson et al.^4^/UKBB^53,54^; MRC IEU ID: ieu-b-110^44,57^; https://gwas.mrcieu.ac.uk/datasets/ieu-b-110/	Circulating, non-fasted measure of LDL; mean age for participants of 57	440,546
eQTLs (in blood)	eQTLGen Consortium by Võsa et al.^43^; https://www.eqtlgen.org/index.html	*cis*-eQTLs (SNP-gene <1Mb distance from center of gene)	up to 16,987
*APOB* gene expression in 13 brain regions, liver, and small intestine	Gene-Tissue Expression Project (GTEx)^59^ https://gtexportal.org/home/datasets	RNA-seq (in transcripts per million) by tissue	Average across brain regions, liver, and small intestine = 204

All GWA studies were performed in those of European ancestry and on males and females.

*GWA* genome-wide association, *eQTL* expression quantitative-trait loci, *NMR* nuclear-magnetic resonance, *TSS* transcription start site, *MRC IEU* Medical Research Council Integrative Epidemiology Unit (University of Bristol).

### MR screen of 103 circulating NMR-measured metabolites on healthspan

**Fig. 3:**
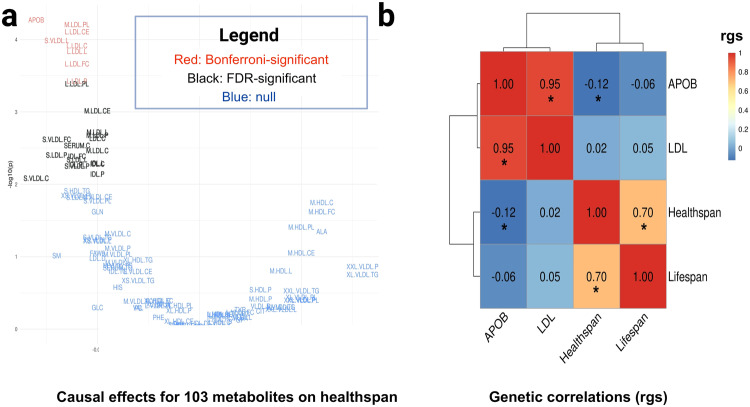
Volcano plot for the MR screen of metabolites on healthspan and heatmap of genetic correlations between (parental) lifespan, healthspan, apolipoprotein B (APOB), and low-density lipoprotein cholesterol (LDL). **a** Volcano plot of the inverse-variance weighted (IVW) betas (X-axis) from the MR screen of 103 metabolites on healthspan. The metabolites consist of lipoproteins, lipids, small molecules, and amino acids. See Supplementary Data 3 for the metabolite names and units, which were provided by Kettunen et al., (2016). The size category is given first, if applicable: e.g., XL = extra-large, L = large, M = medium, S = small and XS = extra-small. Next, the lipoprotein particle is given: VLDL = very-low-density lipoprotein particle, IDL = intermediate-density lipoprotein particle, LDL = low-density lipoprotein particle, and HDL = high-density lipoprotein particle. Last, the lipid measure of the particle is given: C = total cholesterol, D = the mean diameter of the particle, FC = free cholesterol, L = total lipids, P = particle concentration, PL = phospholipids, TG = triglycerides^21^. In the upper-left quadrant are the Bonferroni-significant metabolites (in red): APOB and various lipoproteins containing LDL decrease healthspan. Similarly, in green are the false-discovery rate (FDR)-significant metabolites, of which most are LDL species which also decrease healthspan. **b** Genetic correlations between longevity measures (healthspan and lifespan) with APOB and LDL. Asterisk indicates Bonferroni-level significance (0.05/6 = *P* < 0.008). Strong positive correlation between lifespan and healthspan (*rg* = 0.70) and an inverse correlation between healthspan and higher levels of APOB. The genetic correlation between healthspan and LDL was null.

We began our investigation testing whether metabolites influence healthspan. To do this, we (a) extracted metQTLs data for a set of 103 circulating metabolites (lipoproteins, lipids, small molecules, and amino acids) measured with an NMR platform and (b) extracted the SNP data (i.e., summary statistics) for the same metQTLs from within a GWA study of healthspan^22^. Using these data, we performed an inverse-variance weighted (IVW) MR screen (comprising meta-analyses with ≥ three independent SNPs per metabolite: those not in linkage disequilibrium [LD], *r* < 0.001) of the metabolites in relation to healthspan. To account for multiple testing, we applied a Bonferroni-correction for the 103 IVW meta-analyses (Fig. 3a). Nine Bonferroni-significant metabolites shortened healthspan: apolipoprotein B (APOB) and eight lipoproteins including LDL. The eight lipoproteins including LDL included: cholesterol in large LDL (L.LDL.C), cholesterol esters in large LDL (L.LDL.CE), free cholesterol in large LDL (L.LDL.FC), total lipids in large LDL (L.LDL.L), concentration of large LDL particles (L.LDL.P), phospholipids in large LDL (L.LDL.PL), phospholipids in medium LDL (M.LDL.PL), and total lipids in small VLDL (S.VLDL.L). A comparison of the IVW estimates for the top metabolites with three sensitivity estimators (MR-Egger, weighted median, and weighted mode methods)—a qualitative screen against pleiotropy in the IVW estimate^23^—revealed that the meta-analytic estimators aligned in their directions and magnitudes of effect for APOB and the eight LDL-containing lipoprotein measures. The MR-Egger intercept test was also performed as a formal test against unbalanced pleiotropy in the IVW estimator for the top findings. Together, these sensitivity tests provided no evidence for horizontal pleiotropy in the IVW estimates for the top metabolites in the screen. Supplementary Data 2–4 display the metabolite-screen results in tabular form, provide the SNP characteristics (Supplementary Data 3), show details of the sensitivity analyses for horizontal pleiotropy using the MR-Egger intercept test, and provide the results for the MR-Egger, weighted median, and weighted mode sensitivity estimators. The metabolite screen yielded evidence for a shortening of healthspan per standard-deviation (SD)-unit increases in levels of APOB and various LDL-containing lipoproteins.

### Genetic correlations between APOB, LDL, parental lifespan, and healthspan

Next, to triangulate the MR analyses with a different genetic approach, we calculated genetic correlations between APOB, LDL, and two measures of longevity (parental lifespan [hereafter “lifespan”] and healthspan).

We used linkage disequilibrium score regression (LDSC)^24^ to calculate the genetic correlations (i.e., shared genetic architectures). While LDSC is not a causal inference method (being rather a correlative measure), it is complementary to two-sample MR in that it is not biased by sample overlap^24^ (participants of the GWA studies being in both studies under investigation). LDSC revealed that healthspan (SNP-heritability [${h}_{g}^{2}$] = 0.03) and lifespan (${h}_{g}^{2}$ = 0.02) were strongly positively genetically correlated (genetic correlation [*rg*] = 0.70; *P* = 5.27E-51) (Fig. 3b; Supplementary Data 5). This demonstrates the extensively shared genetic architecture between healthspan and lifespan but also suggests independent genetic components for each trait (0.30 not genetically correlated). Healthspan and APOB (${h}_{g}^{2}$ = 0.09) were negatively genetically correlated (*rg* = −0.12; *P* = 1.26E-03). The relationship between healthspan and LDL (${h}_{g}^{2}$ = 0.08) was null (*rg* = 0.02; *P* = 0.68). We also looked at lifespan and APOB and lifespan and LDL. The relationship between lifespan and APOB was null (*rg* = −0.06; *P* = 0.07). Likewise, the relationship between lifespan and LDL was null (*rg* = 0.05; *P* = 0.21). APOB and LDL were strongly genetically correlated (*rg* = 0.95; *P* < 5E-51). The genetic associations between healthspan and lifespan, healthspan and APOB, and APOB and LDL were Bonferroni-significant (0.05/6 = 8.33E-03).

### Sensitivity tests for the MR of NMR-measured APOB on healthspan

**Fig. 4:**
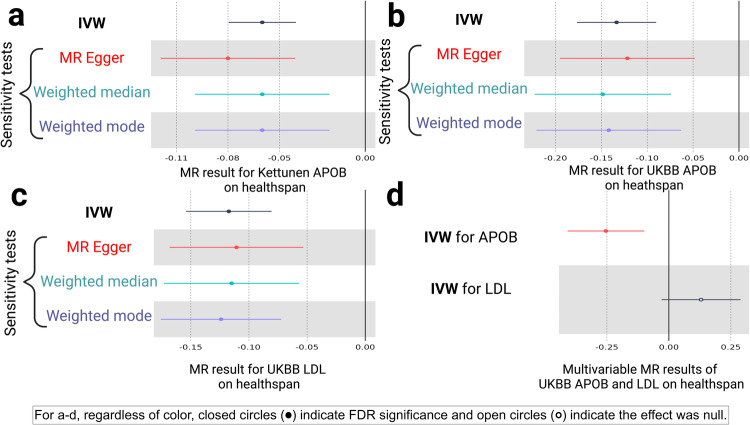
Forest plots of the univariate and multivariable Mendelian randomization (MR) tests of APOB and LDL on healthspan. Forest plots illustrating (**a**) the MR results for nuclear magnetic resonance (NMR)-measured APOB (Kettunen et al., 2016) on healthspan, (**b**) UK Biobank (UKBB) APOB on healthspan, (**c**) UKBB LDL on healthspan, and (**d**) the multivariable MR analysis of UKBB APOB and UKBB LDL on healthspan. For (**a**−**c**), in black are the inverse-variance weighted (IVW; main MR test) and sensitivity estimators (MR-Egger [red], weighted median [cyan], and weighted mode [purple]). The error bars correspond to 95% confidence intervals. The solid-black, vertical lines indicate the null of beta = 0. Solid circles indicate *P* < 0.05. The direction and magnitude of the meta-analytic estimators are compared as screen for pleiotropy: if they align, this is evidence against pleiotropy in the IVW estimate. For (**a**−**c**), the IVW estimate was <0 and the confidence intervals did not cross zero, indicating that Kettunen APOB, UKBB APOB, and UKBB LDL shorten healthspan. Also, for (**a**−**c**), the sensitivity estimators aligned in their directions and magnitudes with their respective IVW estimates, indicating no evidence for horizontal pleiotropy in the IVW estimate. **d** Multivariable MR analysis reveals that when accounting for LDL, APOB remains negatively associated with healthspan (it shortens it). When accounting for APOB, the effect of LDL on healthspan is null.

After this, we performed additional sensitivity analyses for the NMR-measured APOB and healthspan (Supplementary Data 4), though not for the significant LDL-containing lipoprotein measures in relation to healthspan. This was due to a lack of genetic correlation between the LDL and healthspan (Fig. 3b). The additional sensitivity analyses for the MR of NMR-measured APOB on healthspan included RadialMR to detect and remove outliers, as outliers can be a source of pleiotropy in multi-variant genetic instruments. Three outliers were removed (Supplementary Data 2), and the IVW, MR-Egger, weighted median, and weighted mode estimators were run with a 10-SNP NMR-measured APOB instrument. This final instrument for the MR test of NMR-measured APOB on healthspan produced the following results: IVW (estimate = −0.06; 95% confidence interval [CI]: −0.09, −0.03; *P* = 7.56E-06). The meta-analytic estimators aligned in their directions of effect and differed only slightly in their magnitudes: MR-Egger (estimate = −0.08; 95% CI: −0.12, −0.03; *P* = 1.29E-02), weighted median (estimate = −0.06; 95% CI: −0.10, −0.03; *P* = 8.72E-04); weighted mode (estimate = −0.06; 95% CI: −0.10, −0.02; *P* = 9.91E-03). The proportion of variance in NMR-measured APOB explained by the SNP instrument (r2) was 0.06. The *F*-statistic, a measure of instrument strength for the IVW, was 101 (*F*-statistics > 10 are conventionally deemed acceptable^25^), and *I*-squared for the MR-Egger (${I}_{{GX}}^{2}$) for testing the “NO Measurement Error” (NOME) assumption was 0.94 (${I}_{{GX}}^{2}$ > 0.90 is conventionally acceptable^26^). The MR-Egger intercept test suggested no evidence of horizontal pleiotropy in the IVW estimate (MR-Egger intercept = 0.003; *P* = 0.46). Cochrane’s test for heterogeneity was null (*Q*-statistic for the IVW estimate = 8.55, degrees of freedom (df) = 9, *P* = 0.48). The final NMR-measured APOB results are displayed in Fig. 4a.

### MR replication of UK Biobank (UKBB) APOB and LDL on healthspan

The metabolite screen above for the 103 NMR-measured metabolites was performed in only ~24,925 participants. While this sample size is reasonable for a screen, we sought to replicate the top findings (APOB and LDL) with larger instrumental variable data sources. To do this, we used the UKBB as the replication cohort, which has 439,214 participants for a measure of APOB (g/L) and 440,546 participants for a measure of LDL (mmol/L) (both GWA studies performed by Richardson et al. ^4^ Table 1). After removing outliers with RadialMR (viewable in Supplementary Data 6 and 7), we created a 91-SNP instrument for APOB (r2 = 0.02; *F*-statistic = 123; ${I}_{{GX}}^{2}$ = 0.97) and a 90-SNP instrument for LDL (r2 = 0.03; *F*-statistic = 171; ${I}_{{GX}}^{2}$ = 0.98).

For the MR of UKBB APOB on healthspan, the MR-Egger intercept test suggested no evidence of horizontal pleiotropy (MR-Egger intercept = −0.0004; *P* = 0.70). Cochrane’s test heterogeneity was null (no evidence of heterogeneity; *Q*-statistic for the IVW estimate = 82, df = 90, *P* = 0.71). The IVW estimate and meta-analytic estimators replicated the findings from the MR test of NMR-measured APOB, indicating that higher levels of APOB shorten healthspan (IVW estimate = −0.13; 95% CI: −0.18, −0.09; *P* = 1.38E-09; MR-Egger estimate = −0.12; 95% CI: −0.20, −0.05; *P* = 1.63E-03; weighted median estimate = −0.15; 95% CI: −0.22, −0.07; *P* = 9.34E-05; weighted mode estimate = −0.14; 95% CI: −0.22, −0.06; *P* = 6.65E-04). The MR-Egger (−0.12), weighted median (−0.15), and weighted mode (−0.14) estimators aligned in their directions and mostly in their magnitudes with the IVW (−0.13) (Fig. 4b).

Similarly, for LDL, the MR-Egger intercept suggested no evidence of horizontal pleiotropy (MR-Egger intercept = −0.0003; *P* = 0.77). Cochrane’s test for heterogeneity was null (*Q*-statistic for the IVW estimate = 86, df = 89, *P* = 0.57). The IVW estimate and meta-analytic estimators revealed the following: higher levels of LDL shorten healthspan (IVW estimate = −0.12; 95% CI: −0.15, −0.08; *P* = 4.08E-10; MR-Egger estimate = −0.11; 95% CI: −0.17, −0.05; *P* = 2.89E-04; weighted median estimate = −0.11; 95% CI: −0.17, −0.06; *P* = 1.04E-04; weighted mode estimate = −0.12; 95% CI: −0.18, −0.07; *P* = 9.85E-06) (Fig. 4c). The MR-Egger (−0.11), weighted median (−0.11), and weighted mode (−0.12) estimates aligned in their directions and magnitudes with the IVW (−0.12). These observations suggest that higher levels of APOB and LDL shorten healthspan.

### Multivariable MR of APOB and LDL on healthspan

Having observed with univariate MR analyses that higher levels of APOB and LDL shorten healthspan, we performed a multivariable MR analysis of APOB and LDL on healthspan. The benefit of multivariable MR is that it yields direct (versus total) effects, enabling the assessment of the effect of LDL when accounting for APOB and the effect of APOB when accounting for LDL, which is biologically informative since APOB is the main lipoprotein in LDL^3^. When accounting for the effect of APOB, LDL was null (estimate = 0.13; 95% CI: −0.03, 0.29; *P* = 0.11). When accounting for the effect of LDL, APOB continued to reflect that elevated APOB shortens healthspan (estimate = −0.25; 95% CI: −0.41, −0.09; *P* = 0.001; Fig. 4d; Supplementary Data 8). This analysis further supports APOB as a key metabolite influencing healthspan.

### MR of UKBB APOB on AD

**Fig. 5:**
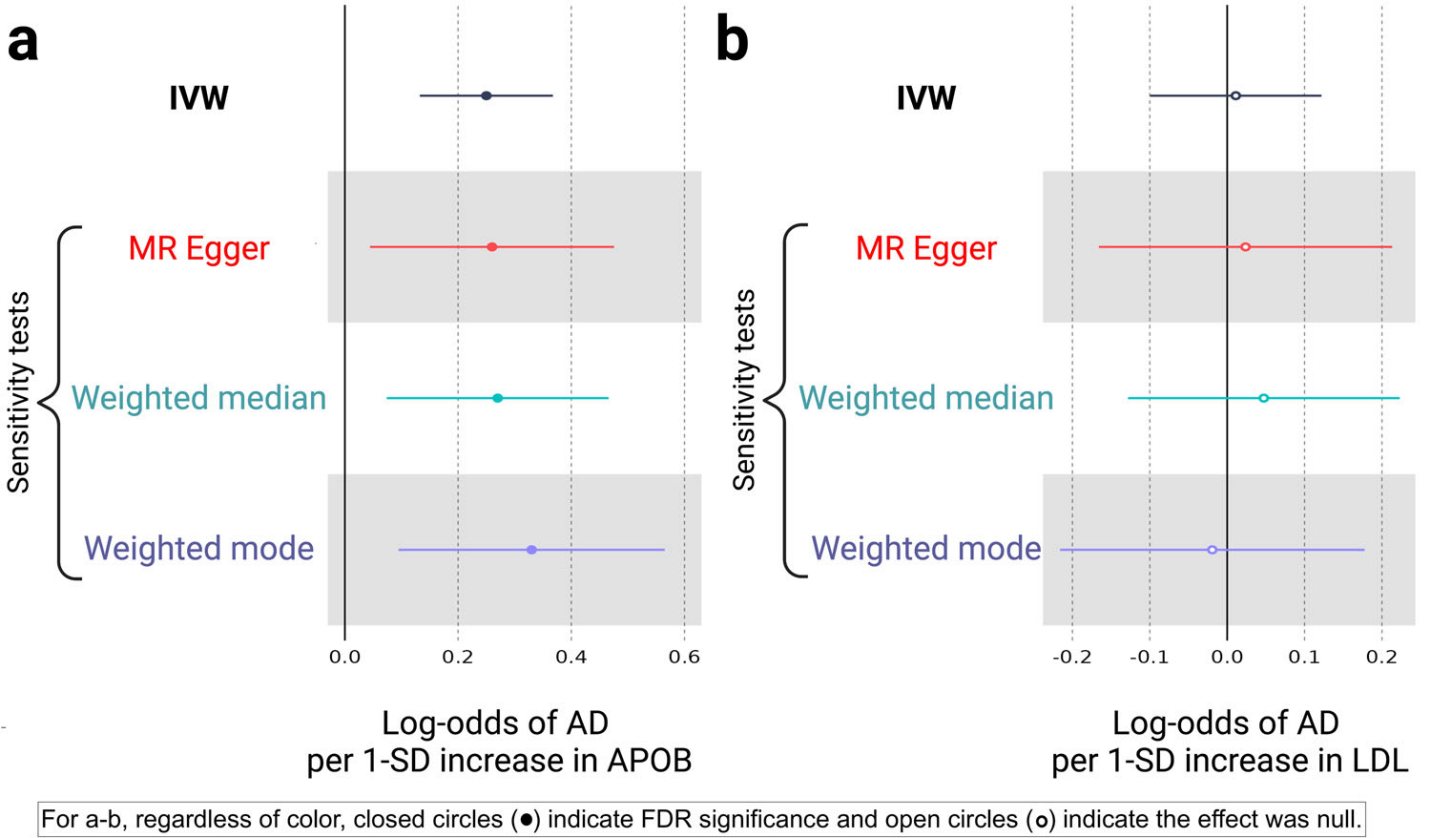
Forest plots illustrating the Mendelian randomization (MR) results for UK Biobank (UKBB) apolipoprotein B (APOB) on Alzheimer’s disease (AD) and UKBB low-density lipoprotein cholesterol (LDL) on AD. In black are the inverse-variance weighted (IVW; main MR test) and sensitivity estimators (MR-Egger [red], weighted median [cyan], and weighted mode [purple]). The error bars correspond to 95% confidence intervals. The solid-black, vertical lines indicate the null. Solid circles indicate *P* < 0.05. Results are displayed on the log-odds scale. **a** For the MR of UKBB APOB on AD, the IVW estimate was >0, and the confidence intervals did not cross zero, indicating that APOB increases risk for AD. Also, the direction and magnitude of the meta-analytic estimators tightly align, which is qualitative evidence against unbalanced pleiotropy in the IVW estimate. **b** The results for the MR of UKBB LDL on AD are null: the IVW (and meta-analytic) estimates hover around zero, and their confidence intervals cross it.

Having gained evidence that APOB shortens healthspan, we aimed to discover whether APOB increases risk for a condition that terminates healthspan: AD. The GWA study for AD was conducted by the International Genomics of Alzheimer’s Project (IGAP) in a discovery sample containing 21,982 cases and 41,944 controls (Table 1). For APOB, there were 131 (vs 91 for the MR of APOB on healthspan) instrumental SNPs available after removing outliers with RadialMR (r2 = 0.03; *F*-statistic = 105; ${I}_{{GX}}^{2}$ = 0.96; Supplementary Data 9 contains a list of removed outliers). The MR-Egger intercept test suggested no evidence for horizontal pleiotropy (MR-Egger intercept = −0.0003; *P* = 0.93). Cochrane’s test heterogeneity was null (*Q*-statistic for the IVW estimate = 116, df = 130, *P* = 0.81). The IVW estimate and meta-analytic estimators indicated that higher levels of APOB increase risk for AD (IVW estimate [log odds] = 0.25; 95% CI: 0.13, 0.37; *P* = 3.94E-05; MR-Egger [log odds] = 0.26; 95% CI: 0.04, 0.48; *P* = 1.2.25E-02; weighted median [log odds] = 0.27; 95% CI: 0.08, 0.47; *P* = 5.15E-03; weighted mode [log odds] = 0.33; 95% CI: 0.09, 0.57; *P* = 8.07E-03). The MR-Egger (0.26), weighted median (0.27), and weighted mode (0.33) estimators aligned in their directions and mostly in their magnitudes with the IVW (0.25) (Fig. 5a).

### Partial replication of MR of UKBB APOB on AD

Having gained evidence that higher levels of APOB influence risk for AD, we sought a partial replication. The replication is partial because the AD GWA summary statistics for the replication (performed by Jansen et al. ^27^) included some of the participants in the IGAP AD GWA study. The advantage of using the Jansen GWA data, however, is that it included 71,880 cases (some clinically defined and some “proxy” cases; see *Methods*). Thus, Jansen’s summary statistics contained more than triple the number of AD cases as IGAP alone. We created a 135-SNP instrument after removing outliers with RadialMR (r2 = 0.05; *F*-statistic = 161; ${I}_{{GX}}^{2}$ = 0.99; Supplementary Data 10). The IVW estimate and meta-analytic estimators comported with the MR of APOB on IGAP AD, showing that higher levels of APOB increase risk for AD, though the magnitude of the effect attenuated (IVW estimate [log odds] = 0.02; 95% CI: 0.01, 0.04; *P* = 1.44E-03; MR-Egger [log odds] = 0.01; 95% CI: −0.01, 0.04; *P* = 2.21E-01; weighted median [log odds] = 0.01; 95% CI: −0.01, 0.03; *P* = 3.83E-01; weighted mode [log odds] = 0.01; 95% CI: −0.01, 0.04; *P* = 1.91E-01).

### MR of UKBB LDL on AD

Next, we sought to ascertain whether LDL increased risk for AD. For LDL, there were 121 (vs 90 for the MR of LDL on healthspan) instrumental SNPs available after removing outliers with RadialMR (r2 = 0.04; *F*-statistic = 138; ${I}_{{GX}}^{2}$ = 0.98; Supplementary Data 11 contains a list of removed outliers). The MR-Egger intercept test suggested no evidence for horizontal pleiotropy (MR-Egger intercept = −0.0005; *P* = 0.87). Cochrane’s test for heterogeneity was null (*Q*-statistic for the IVW estimate = 106, df = 121, *P* = 0.84). The IVW estimate and meta-analytic estimators were null, indicating that higher levels of LDL do not increase risk for AD (IVW estimate [log odds] = 0.01; 95% CI: −0.10, 0.12; *P* = 0.84; MR-Egger [log odds] = 0.02; 95% CI: −0.17, 0.21; *P* = 0.81; weighted median [log odds] = 0.05; 95% CI: −0.13, 0.22; *P* = 0.60; weighted mode [log odds] = −0.02; 95% CI: −0.20, 0.16; *P* = 0.84). See Fig. 5b. Since the univariable MR of LDL on AD was null, we did not perform a multivariable MR analysis of APOB and LDL on AD.

Next, we examined the genetic instrument for APOB more closely. If the SNPs in the genetic instrument for APOB are also associated with another trait that increases risk for AD, this could possibly violate the MR assumption that the genetic instrument for APOB influences AD only through APOB levels (Fig. 2b). Mutations in *APOE* are known risk factors for AD. We looked up APOE in PhenoScanner, a curated database of GWA study results that cross-references genetic variants with a broad array of phenotypes^28,29^. We did this to ascertain whether any of the SNPs associated with APOE were also associated with APOB. Two SNPs on chromosome 19 were found to be associated with both APOE and APOB levels: rs7412 and rs7249565. Neither of these SNPs were used as genetic instruments for APOB in the MR of APOB on AD. However, knowledge of whether our genetic instrument for APOB contained SNPs on chromosome 19 in LD with rs7412 and rs7249565 was not yet known to us. To decipher whether this was the case, we used LDlink, a web-based tool to interrogate LD^30^. None of SNPs in our instrument for APOB on chromosome 19 were in LD with either rs7412 or rs7249565 (Supplementary Data 12).

### Blood-based summary-data based MR (SMR) of APOB

**Fig. 6:**
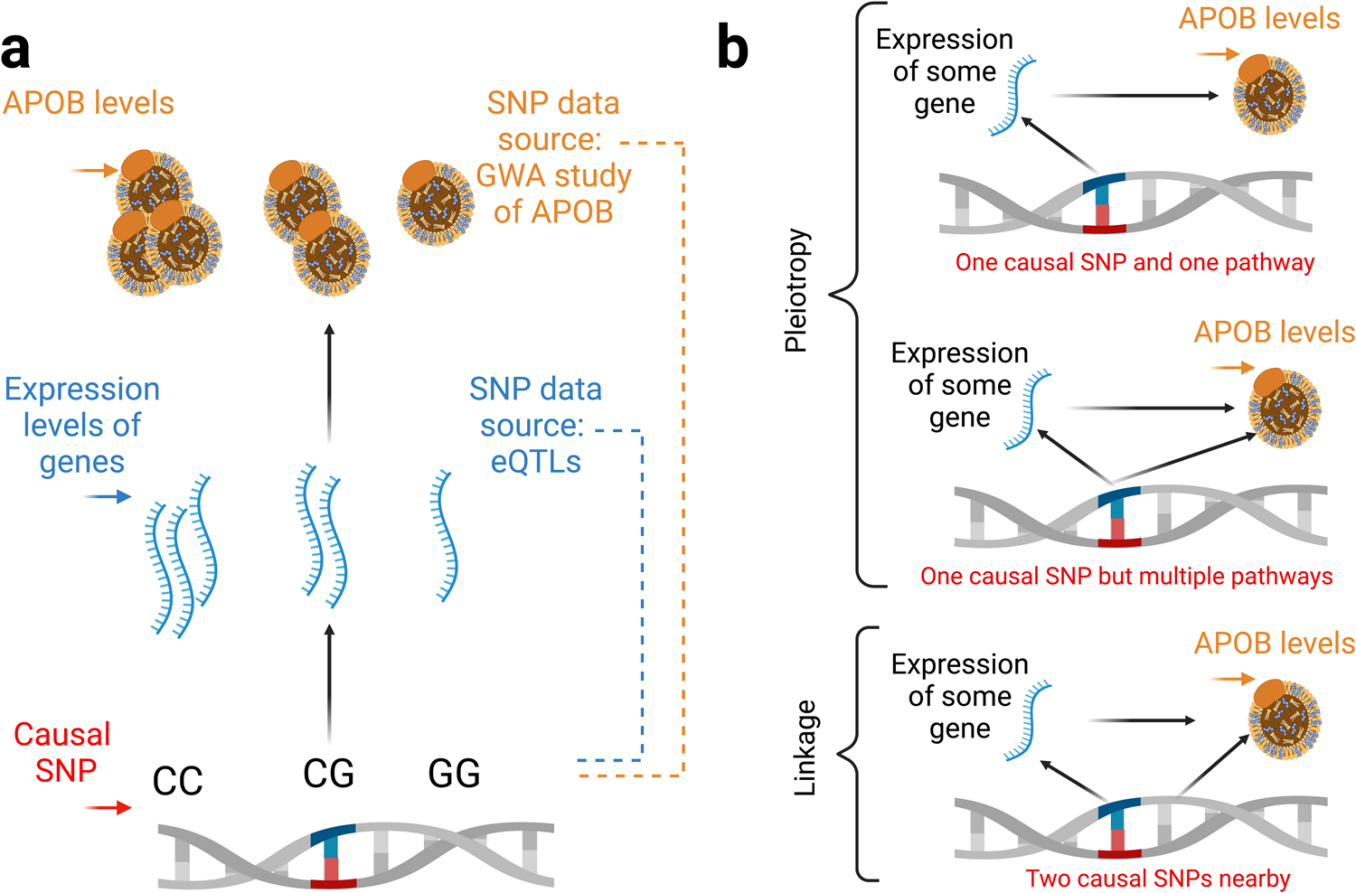
Conceptual framework for transcriptomic summary-data based Mendelian randomization (SMR) of apolipoprotein B (APOB). **a** This is a model representing differences in APOB levels that are caused by the dose of a single-nucleotide polymorphism (SNP)’s effect allele on expression and subsequently APOB protein levels. SMR is a method that can use expression quantitative-trait loci in models instead of gene expression. **b** Three explanations for a significant SMR association are depicted: causal pleiotropy (the SNP influences expression and levels of APOB through a single causal pathway); horizontal pleiotropy (the SNP influences expression of some gene and APOB levels, but the SMR result is due to the SNP’s effect on another process); and genetic architecture (i.e., linkage), where there are two underlying SNPs near each other in the genome, one impacting gene expression and the other impacting APOB levels. With SMR alone, it is not possible to distinguish causal from horizontal from pleiotropy. But it is possible to assume a single underlying SNP is responsible for an SMR signal (i.e., to assume pleiotropy vs. linkage). Procedures for doing so are called “genetic colocalization.” We filtered for genetic colocalization with the heterogeneity in dependent instruments (HEIDI) method by Zhu et al.^61^ to reduce false-positive findings^61^. Figure inspired by Zhu et al.^61^.

Having observed that APOB impacts healthspan and increases risk for AD, we sought to identity genes whose expression in blood influences APOB concentrations. To do so, we integrated expression quantitative-trait loci (eQTL) data with the UKBB GWA study data for APOB. A conceptual approach for this presented in Fig. 6.

**Fig. 7:**
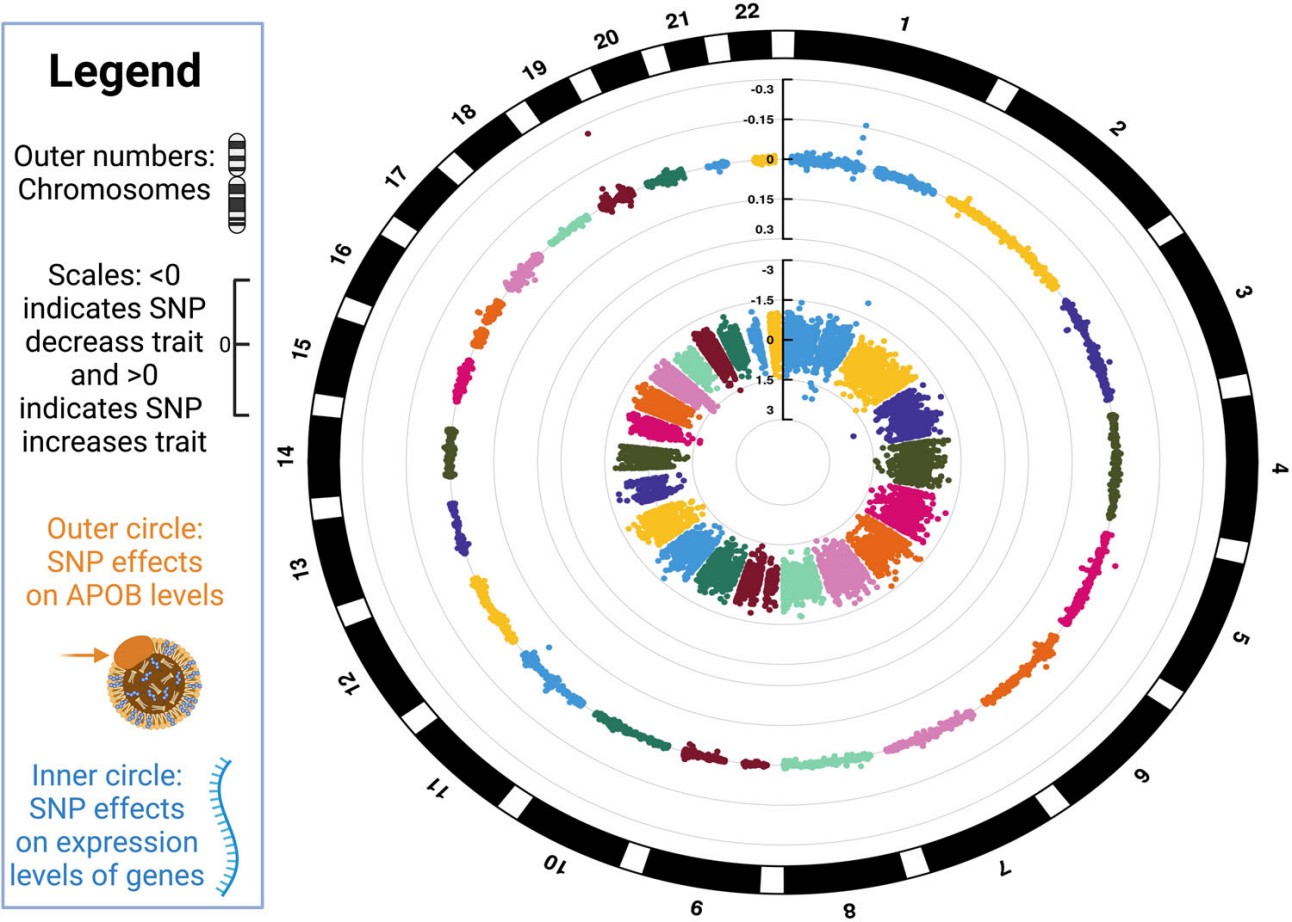
Circos plot of the data for the summary-data Mendelian randomization (SMR) of genes whose expression influences apolipoprotein B (APOB) levels. The plot depicts SNP effects on *APOB* (outer circle) and gene expression (inner circle). The vertical bars in the center with ranges indicate SNP effects. Values >0 indicate an increase in APOB (outer circle) and an increase in expression (inner circle). Conversely, values <0 indicate a decrease in APOB (outer circle) and a decrease in expression (inner circle). These are the data for summary-data based Mendelian randomization (SMR). 14,057 lead eQTLs (*P* < 5 x 10^-6^) were testable in relation to circulating APOB levels. We used these data to obtain the effect of gene expression on APOB levels by calculating Wald ratios for individual SNPs (i.e., dividing a SNP’s effect on APOB by a SNP’s effect on gene expression). See Supplementary Data 14 for detailed SNP characteristics. The circos plot was created with CMplot^62^.

We identified 14,057 (lead) eQTLs (*P* < 5 x 10^-6^) in blood that were testable in relation to circulating APOB concentrations. We used the data in Fig. 7 to obtain the effect of gene expression on APOB by calculating Wald ratios (i.e., dividing a SNP’s effect on APOB by a SNP’s effect on gene expression). See Supplementary Data 13 for specific SNP characteristics (e.g., the effect of the SNP on gene expression and the effect of the SNP on APOB).

After Bonferroni correction to account for multiple testing, heterogeneity of instruments (HEIDI) filtering for genetic colocalization (HEIDI *P* > 0.05), and *F*-statistic filtering (*F* > 10), there were 49 genes whose expression in blood appeared to influence circulating APOB concentrations (Supplementary Data 14). However, we further controlled for false-positive associations by restricting our top findings to those with Bonferroni-corrected SMR *P* < 5E-8. After this, 10 genes remained. We performed Bayesian genetic colocalization (coloc^31^) on this set of 10 genes (Table 2 and Supplementary Data 15). Bayesian genetic colocalization estimates a posterior probability that a single genetic variant affects both traits. Conventionally, a posterior probability (PP4) > 0.80 is considered evidence for this^32^. Although HEIDI suggested evidence of no heterogeneity between instruments for the 10 genes, only two had posterior probabilities >0.80: *PELO* (pelota mRNA surveillance and ribosome rescue factor) and *PYGB* (glycogen phosphorylase, brain). Notably, Bayesian genetic colocalization identified the lead SNP used by HEIDI as the likely causal SNP in both cases: *PELO* (rs1499279) and *PYGB* (rs11699953), increasing confidence that *PELO* and *PYGB* are potential drug targets to modify APOB concentrations.

**Table 2:** Top findings for the summary data-based Mendelian randomization (SMR) of expression in blood on circulating APOB concentrations

Gene	Top SNP	Top SNP Chr	Top SNP bp	Effect allele	Other allele	SMR effect estimate	SMR *P*	Coloc region (500kb +/− lead SNP from SMR)	No. Coloc SNPs	PP4	Causal SNP
*PELO*	rs1499279	5	52106278	G	T	0.004	4.52E-27	5:51606278-52606278	2860	9.88E-01	rs1499279
*RASIP1*	rs1231281	19	49239200	A	G	0.043	2.33E-12	19:48739200-49739200	2726	1.61E-44	NA
*ILRUN*	rs3800461	6	34616322	C	G	0.007	2.52E-12	6:34116322-35116322	1958	2.93E-13	NA
*SARM1*	rs4795434	17	26716917	G	T	0.010	3.05E-11	17:26216917-27216917	1097	5.19E-09	NA
*GRINA*	rs57957974	8	145076529	A	C	0.004	6.77E-11	8:144576529-145576529	1544	1.93E-13	NA
*SKAP1*	rs34791545	17	46488447	C	T	0.007	3.14E-10	17:45988447-46988447	1939	5.09E-10	NA
*ZNF664*	rs7958691	12	124440743	T	G	0.013	4.68E-10	12:123940743-124940743	2192	7.89E-12	NA
*PARP10*	rs11784833	8	145063412	C	T	0.010	5.05E-10	8:144563412-145563412	1561	1.93E-13	NA
*PYGB*	rs11699953	20	25241345	G	C	0.003	9.40E-09	20:24741345-25741345	2696	9.14E-01	rs11699953
*EVI2B*	rs9902893	17	29625638	A	G	0.023	1.01E-08	17:29125638-30125638	1829	1.27E-04	NA

*SNP* single-nucleotide polymorphism, *Chr* chromosome, *Bp* top SNP base pair. Displayed SMR findings had heterogeneity in dependent instruments (HEIDI) *P* > 0.05 and *F*-statistics > 10 (Supplementary Data 14). *SMR P* Bonferroni-corrected *P*-value for the SMR test. *Coloc* Fully Bayesian Genetic Colocalization using Bayes Factors. PP4 = posterior probability that a single genetic variant affects both traits (PP4 > 0.80 suggests evidence that a single variant is responsible for the eQTL and apolipoprotein B (APOB) signal^32^).

## Discussion

Our MR screen identified APOB and lipoproteins containing APOB as causal determinants of healthspan, a finding that is concordant with recent observations regarding APOB and lifespan^1,2^. Our findings for APOB and LDL in relation to healthspan replicated using the UKBB genetic instruments and remained robust when subjected to a battery of sensitivity analyses. In addition, multivariable MR of APOB and LDL on healthspan revealed that the impact of APOB on healthspan is partially independent of LDL: APOB remains significant when accounting for LDL levels as a confounder. Genetic correlation analysis suggested that APOB (but not LDL) shortens healthspan. Together, these lines of evidence strengthen the hypothesis that APOB is implicated in longevity via modulation of healthspan and build on the findings by Perrot et al. (2020)^2^ and Richardson et al. (2021)^1^. Like us, Perrot et al. ^2^ performed MR analyses of circulating metabolites on lifespan using the Kettunen^21^ metabolite instruments. They found evidence that higher APOB and APOB-containing lipoproteins shorten lifespan. We extend their observation about APOB and longevity by showing that APOB shortens healthspan. Moreover, we also extend the work by Richardson et al.^1^, who observed that APOB is implicated in type 2 diabetes. Like AD, type 2 diabetes ends healthspan.

In addition, we found that circulating APOB (but not LDL) increases risk for AD. This finding for APOB in relation to AD conflicts with that by Williams et al. (2020)^33^, who sought to identify genetic support for the repurposing of mipomersen (an antisense oligonucleotide inhibitor of APOB^34^) for AD prevention. Like us, Williams and colleagues used IGAP GWA study data for the AD outcome source in their two-sample MR. Their study differed from ours in the construction of their genetic instrument, though. Whereas our instruments for APOB contained more SNPs, which were clumped to prevent LD, they used a principal-components-based MR method. We attempted a partial replication of our MR of APOB on AD using a substantially larger GWA study containing more than triple of the number of AD cases. Although our partial replication succeeded in the sense that the results revealed that higher levels of APOB increased risk for AD, the effect estimate attenuated towards the null. Future MR studies of AD, ideally with a larger number of clinically defined cases (and not including IGAP cases) are needed to confirm whether the relationship between APOB and AD is causal.

**Fig. 8:**
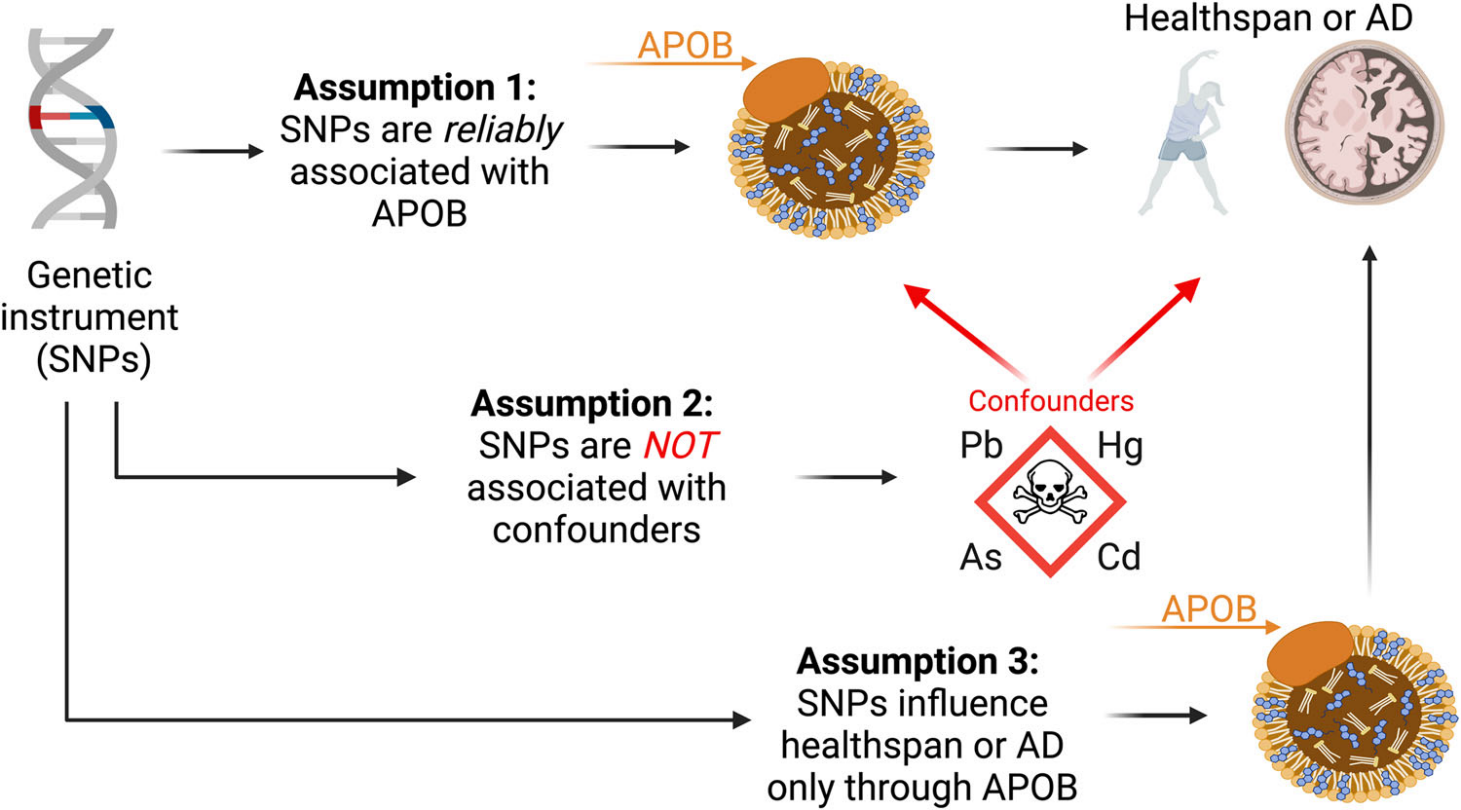
Mendelian randomization assumptions. Depiction of the three main MR assumptions: (1) Genetic instruments (i.e., single-nucleotide polymorphisms [SNPs]) must be reliably associated with apolipoprotein B (APOB). (2) The SNPs for APOB must not be associated with confounders of the APOB-Alzheimer’s disease (AD) or APOB-healthspan relationship. Examples of confounders are depicted as toxic metals and metalloids: lead, Pb; mercury, Hg; arsenic, As; and cadmium; Cd. When this assumption is met, MR avoids most sources of non-genetic confounding. (3) The SNPs for APOB must influence healthspan or AD only through APOB.

The primary strength of our study is a feature of MR generally: quasi-randomization. When certain assumptions are met, MR can facilitate the assessment of causal relationships in studies where RCTs are infeasible^20^. Due to the use of genetic variants as instrumental variables, we avoided most sources of confounding by non-genetic factors. For example, one might wonder whether environmental exposures, such as heavy metals, are responsible for the association between circulating APOB and AD. That is, maybe heavy metals are a confounder of the APOB-AD relationship. This is a realistic concern in observational designs investigating APOB and AD. The reason is that heavy metals have been reported to dysregulate lipoproteins^35,36^, cross the BBB, and cause neurotoxicity^37^. However, with MR, this scenario is improbable since biomarkers of exposure to heavy metals are unlikely to be associated with the SNPs that control APOB (Fig. 8).

Our study is not without limitations. First, using populations of the same continental ancestry is required for both GWA data sets in two-sample MR. Since appropriate GWA sources for metabolites, healthspan, and AD were only available for non-Hispanic whites, our analysis is limited to those of European ancestry. This means our findings may not generalize to those of other ancestries. But even likelier is that we are missing part of the story about APOB. As the data become available, future studies ought to examine the relationships between APOB and healthspan and APOB and AD in other populations.

Second, the MR analyses of UKBB APOB and UKBB LDL on healthspan could possibly suffer to some extent from Winner’s curse. Winner’s curse is an idea stolen from bidding at auctions. It refers to the situation when a bidder wins an item by placing a bid exceeding the item’s value^38^. The bid may be won, but the buyer pays more than the item is worth. Something similar occurs in GWA studies. Namely, sometimes the effect of a causal SNP is overestimated in the discovery GWA study. This can lead to bias in MR approaches that use the data from discovery samples. However, a recent empirical examination of this bias found that it might not practically change the inference in MR^38^. Even though this concern may be minimal, we took measures to avoid Winner’s curse by choosing data sources in which the participants were not expected to overlap. The exceptions are the MR analyses for UKBB APOB and UKBB LDL on healthspan, for which the participants in the healthspan GWA study were also included in the UKBB GWA studies, and the partial MR replication of the UKBB APOB on Jansen AD (which included UKBB participants). As for the MR tests of UKBB APOB and UKBB LDL on healthspan, the MR metabolite screens with NMR-measured APOB and LDL-containing lipids on healthspan function as a sanity check against this issue. This is because the participants in the GWA studies for the metabolite screen (Kettunen et al.) were not in the UKBB, yet the results were comparable to those from the analyses with overlapping samples. Nonetheless, we scrutinized the results for the UKBB APOB on healthspan further by employing MRlap to correct for bias due to weak instruments and Winner’s curse that can be caused by use of overlapping samples in two-sample MR^39^ (see *Methods*). In addition, we made use of LDSC’s intercept for the genetic covariance between UKBB APOB and Jansen AD, which indicated no evidence of sample overlap.

Third, our replication of the APOB and AD MR analysis is partial. Although the results comport with those from the primary analysis, supporting a causal role of APOB on AD, the Jansen et al. ^27^ GWA study included participants in the Kunkle et al. (2019) IGAP GWA study. As such, a true replication, which excludes IGAP participants and includes more cases of AD than IGAP is needed.

A fourth limitation pertains to the SMR results. While HEIDI reduces the likelihood of false-positive associations, it can sometimes fail to detect heterogeneity^40^. This may explain why the Bayesian colocalization approach failed to provide evidence for a single underlying genetic variant for eight of the 10 candidate genes detected by SMR. The low posterior probabilities (PP4) for those eight candidates imply they are unlikely to be useful as drug targets to influence APOB levels. However, use of SMR with HEIDI followed by Bayesian colocalization yielded two promising candidate drug targets whose expression in blood appears to causally influence APOB: *PELO* and *PYGB*.

Ultimately, our findings support the possibility that APOB underlies the causal effects of APOB-containing lipoprotein traits in ending healthspan and increasing the risk for AD. A finer-grained analysis of molecular mechanisms, unfortunately, is beyond our scope but should be part of future research efforts. For now, our results make it considerably more difficult to imagine that maximal strategies for improving healthspan and maintaining optimal cognitive function can ignore APOB.

## Methods

### Data sources

Table 1 contains a list of studies used in the present analysis and web links for accessing the data. All GWA summary statistics were obtained from consortia with participants of European ancestry who made their results public. The original authors obtained informed consent from the participants. Briefly, the lifespan GWA study was reported as a protective ratio (i.e., it was coded by Timmers et al., 2019, to reflect an increase in lifespan)^41^. The healthspan GWA study was reported by Zenin et al. (2019)^22^ as hazard ratio. We converted it to a protective ratio (the negation of the hazard ratio) to make it comparably interpretable with the lifespan GWA study. A such, both measures are interpreted in relation to a prolongation of either life or disease-free living. We obtained the GWA studies for the NMR-measured metabolites from Kettunen et al. (2016)^21^ and the GWA studies for the UKBB measures of APOB and LDL from Richardson et al. (2020)^4^. The GWA data for AD were obtained from IGAP (Kunkle et al., 2019)^42^. The GWA data for the partial replication MR analysis of APOB on AD was obtained from Jansen et al. (2019)^27^, which contained clinically defined cases of AD as well as AD-by-proxy cases. They defined AD-by-proxy cases as individuals with one or two parents with AD in the UKBB. When both parents had AD, they upweighted cases. Proxy controls were defined as those with two parents without AD, where older cognitively normal parents were likewise upweighted, accounting for the higher likelihood of younger parents in the UKBB developing AD later in life. Jansen et al.^27^ included IGAP participants. We obtained eQTL data derived from whole blood in 31,684 individuals from Võsa et al. (2021) of the eQTLGen consortium^43^. We downloaded their binary files, which they had set up for use with SMR. We obtained version 8 data from GTEx Portal: RNA-seq files for gene expression within 13 brain regions, the small intestine (terminal ilium), and liver. The RNA-seq files were normalized by transcript/gene length and provided in transcripts per million.

### Statistics and reproducibility

For the MR screen of 103 metabolites on healthspan, the MR tests of UKBB APOB and LDL on healthspan, and the MR tests of UKBB APOB and LDL on AD, each genetic instrument contained a minimum of three SNPs that were independent (not in LD; *r* < 0.001). Instrumental SNPs were selected at *P* < 0.05 x 10^-8^. Proxy SNPs in LD (r2 = 0.80) with instrumental SNPs were used for UKBB APOB (three proxies; Supplementary Data 9) and UKBB LDL (two proxies; Supplementary Data 11) for the MRs of UKBB APOB and UKBB LDL on AD. We used proxies when instrumental APOB SNPs were not available in the Alzheimer’s GWA study. (We did not use proxy SNPs for the MR tests of UKBB APOB and UKBB LDL on healthspan. This is because we imported the healthspan GWA study into R for use with the TwoSampleMR package, and proxy SNPs were not available using the *extract_outcome_data* function for APOB and healthspan). Wald ratios for the IVW tests were meta-analyzed using first-order weights^44,45^.

For the MR metabolites-screen, a Bonferroni correction was applied to address false positives from multiple testing. We conducted all meta-analytic MR analyses and multivariable MR of UKBB APOB and LDL on healthspan within the TwoSampleMR package^44^ using R version 4.0.3^45^. The multivariable analysis was based on that proposed by Sanderson et al. (2019)^46^.

### Main univariable MR sensitivity analyses

For the NMR-measured APOB on healthspan, the MRs of UKBB APOB and LDL tests on healthspan, and the MRs of UKBB APOB and LDL on AD, RadialMR^47^ was used to detect potential outliers, which were removed. Cochrane’s test for heterogeneity, where a *P* > 0.05 indicates a lack of evidence for heterogeneity in the genetic instrument, was conducted for all univariable tests. Details of the instrument selection are available in Supplementary Data 2, 6, 7, 9, and 11. Sensitivity meta-analyses (i.e., MR-Egger, weighted median, and weighted mode MR) were performed (as reported in *Results*) as qualitative screens for horizontal pleiotropy in the IVW estimator. These qualitative screens for pleiotropy were done in addition to the standard quantitative screen for pleiotropy assessed via the MR-Egger intercept test.

### Genetic correlations

Genetic correlations were calculated between UKBB APOB, UKBB LDL, lifespan, and healthspan using LDSC^24^. We also used LDSC to test for possible sample overlap between the summary statistics for UKBB APOB and Jansen AD by testing whether the intercept for the crosstrait genetic covariance was consistent with zero.

### Post-hoc MR sensitivity analyses

In addition, we performed a battery of post-hoc sensitivity tests to doublecheck for violations of the MR assumptions for the univariable MR tests of APOB and LDL on healthspan and AD. These included removing instrumental SNPs with effects on the outcome GWA study of *P* < 0.05, a potential source of horizontal pleiotropy. We then re-ran the meta-analytic MR models with those SNPs removed. We also implemented the Mendelian Randomization Pleiotropy RESidual Sum and Outlier (MR-PRESSO)^48^ method. MR-PRESSO is a tool for evaluating, detecting, and correcting for horizontal pleiotropy. Whereas RadialMR can be used to detect and remove outliers, MR-PRESSO retains outlier instruments and corrects effect estimates for distortion due to them. In addition, we performed MRlap^39,49^ for the MR of UKBB APOB on healthspan to correct for potential biases due to overlapping samples. Last, we utilized a feature of LDSC—whether the intercept for genetic covariance deviates from zero in a crosstrait analysis—as a sensitivity analysis for sample overlap between the summary statistics for UKBB APOB and Jansen AD. This revealed no evidence of sample overlap despite the possibility for this given both GWA studies using some UKBB samples. Readers are referred to Supplementary Data 16−27 for the results of the post-hoc analyses, as none altered the inferences from the primary analyses.

### SMR

We conducted the blood-based eQTL analyses of genes whose expression influences circulating APOB with SMR (version 1.03). HEIDI was implemented in SMR using LD scores computed from European individuals within 1000 Genomes Project (phase 3)^50,51^. HEIDI is a genetic colocalization method that assumes a single causal variant is responsible for a Wald ratio signal within an LD region. It estimates whether SNPs in an LD region produce Wald ratios more different from each other than expected. Thus, a *P* > 0.05, where the null of a single underlying causal SNP is not rejected, is used as evidence against heterogeneity to reduce false-positive associations.

For the blood eQTL analyses, we selected only *cis*-eQTLs (within 1 megabase [Mb] of a target probe), which reduces the probability of horizontal pleiotropy that is more likely with *trans*-eQTLs. We defined eQTLs based on a threshold of *P* < 5 x 10^-06^. Wald ratios were obtained by dividing the effect estimate for APOB by the effect estimate for the eQTL. To assess instrument strength for the Wald ratios (i.e., the SMR test of expression on APOB), we calculated *F*-statistics by dividing the absolute value of the effect estimate for the eQTL by its standard error^52^. For the blood-based SMR analysis, 10,542 instruments had *F*-statistics >10 (Supplementary Data 13−14). For the genome-wide significant SMR findings (10 genes), we implemented another colocalization procedure: Fully Bayesian Genetic Colocalization using Bayes Factors (coloc; by Wallace and colleagues)^31^. Coloc estimates a posterior probability that a single genetic variant affects both traits. We implemented coloc in R with its default settings for the priors: prior probability a SNP is associated with gene expression (p1 = 1E-4), prior probability a SNP is associated with APOB levels (p2 = 1E-4), and prior probability a SNP is associated with both traits (p12 = 1E-5). We chose to investigate 500 kilobase regions up and downstream of a lead SNP’s position for each candidate gene identified with SMR.

### Reporting summary

Further information on research design is available in the Nature Portfolio Reporting Summary linked to this article.

### Supplementary information

Description of Additional Supplementary Files

Supplementary Data 1

Supplementary Data 2

Supplementary Data 3

Supplementary Data 4

Supplementary Data 5

Supplementary Data 6

Supplementary Data 7

Supplementary Data 8

Supplementary Data 9

Supplementary Data 10

Supplementary Data 11

Supplementary Data 12

Supplementary Data 13

Supplementary Data 14

Supplementary Data 15

Supplementary Data 16

Supplementary Data 17

Supplementary Data 18

Supplementary Data 19

Supplementary Data 20

Supplementary Data 21

Supplementary Data 22

Supplementary Data 23

Supplementary Data 24

Supplementary Data 25

Supplementary Data 26

Supplementary Data 27

Reporting Summary

## Data Availability

All data sources for the analyses undertaken in this study are publicly available (Table 1). The data used to generate Fig. 2b, c are presented in Supplementary Data 1. The data used to create Figs. 4–5 are available in Supplementary Data 4, 6, 7, 8, 9, 11.

## Code availability

Code to run MR analyses can be found at MR-Base (https://mrcieu.github.io/TwoSampleMR/). Code to run LDSC is available at https://github.com/bulik/ldsc. Code to run SMR is available at https://yanglab.westlake.edu.cn/software/smr/#Overview. A tutorial for running Fully Bayesian Genetic Colocalization using Bayes Factors is available by Chris Wallace at https://cran.r-project.org/web/packages/coloc/vignettes/a02_data.html.
