# Supplementary material for: Mendelian randomization reveals apolipoprotein B shortens healthspan and possibly increases risk for Alzheimer’s disease
Source: Commun Biol. 2024 Feb 24;7:230. doi: 10.1038/s42003-024-05887-2 (PMC10894226; doi:10.1038/s42003-024-05887-2)
Supplement: Supplementary file 1 — Description of Additional Supplementary Files [file 42003_2024_5887_MOESM1_ESM.pdf]

Description of Additional Supplementary Files

File name: Supplementary Data 1
Description: Gene-Tissue Expression (GTEx) transcripts per million (TPM) values for apolipoprotein B (APOB) for tissues displayed in Fig. 2.

File name: Supplementary Data 2
Description: Meta -analytic Mendelian randomization (MR) results for 103 nuclear magnetic resonance (NMR)-measured circulating metabolites on healthspan.

File name: Supplementary Data 3
Description: Mendelian randomization (MR) single-nucleotide polymorphism (SNP) data (for calculating the Wald ratios that were meta-analyzed in Supplementary Data 2) for 103 nuclear magnetic resonance (NMR)-measured circulating metabolites on healthspan.

File name: Supplementary Data 4
Description: Detailed meta-analytic Mendelian randomization (MR) results (bottom; below the green bar at line 16; columns W-A0) for Kettunen apolipoprotein B (APOB) (outliers removed) on healthspan.

File name: Supplementary Data 5
Description: Genetic correlations between healthspan, lifespan, low-density lipoprotein cholesterol (LDL), and apolipoprotein (APOB).

File name: Supplementary Data 6
Description: Meta-analytic Mendelian randomization (MR) results and instrument selection (bottom; below the green bar at line 96; columns V-AT) and SNP data (including Wald ratios; above green line) for UK Biobank-measured (circulating, non-fasted) APOB on healthspan.

File name: Supplementary Data 7
Description: Meta-analytic Mendelian randomization (MR) results and instrument selection (bottom; below the green bar at line 95; columns V-AT) and SNP data (including Wald ratios; above green line) for UK Biobank-measured (circulating, non-fasted) LDL on healthspan.

File name: Supplementary Data 8
Description: Multivariable MR results for APOB and LDL on healthspan.

File name: Supplementary Data 9
Description: Meta-analytic Mendelian randomization (MR) results and instrument selection (bottom; below the green bar at line 137) and SNP data (including Wald ratios; above green line) for UK Biobank-measured (circulating, non-fasted) apolipoprotein B (APOB) on Alzheimer's disease.

File name: Supplementary Data 10
Description: Partial replication with meta-analytic Mendelian randomization (MR) results and instrument selection (bottom; below the green bar at line 138) and SNP data (including Wald ratios; above green line) for UK Biobank-measured (circulating, non-fasted) apolipoprotein B (APOB) on Jansen et al. (2019) Alzheimer's disease.

File name: Supplementary Data 11

Description: Meta-analytic Mendelian randomization (MR) results and instrument selection (bottom; below the green bar at line 128) and SNP data (including Wald ratios; above green line) for UK Biobank-measured (circulating, non-fasted) low-density lipoprotein cholesterol (LDL) on Alzheimer's disease.

File name: Supplementary Data 12
Description: Exploration of linkage disequilibrium (r2) between apolipoprotein E (APOE) SNPs also associated with apolipoprotein B (APOB) and the APOB instruments on chromosome 19 used as a genetic instrument for the Mendelian randomization (MR) test of APOB on Alzheimer's disease.

File name: Supplementary Data 13
Description: All summary-data based Mendelian randomization (SMR) results in blood for circulating apolipoprotein B (APOB) levels.

File name: Supplementary Data 14
Description: Bonferroni significant summary-data based Mendelian randomization (SMR) results in blood for circulating apolipoprotein B (APOB) levels.

File name: Supplementary Data 15
Description: Bayesian colocalization (coloc) findings for the top 10-most (genome-wide) significant summary-based Mendelian randomization (SMR) of apolipoprotein B (APOB) results (columns Z-AC).

File name: Supplementary Data 16
Description: Instrument sensitivity analysis removing instruments with outcome p-values <0.05 (outliers detected and removed with RadialMR prior) for the meta-analytic Mendelian randomization (MR) of UK Biobank-measured (circulating, non-fasted) apolipoprotein B (APOB) on healthspan.

File name: Supplementary Data 17
Description: Mendelian Randomization Pleiotropy RESidual Sum and Outlier (MR PRESSO) sensitivity analysis of the meta-analytic MR of UK Biobank-measured (circulating, non-fasted) apolipoprotein B (APOB) on healthspan (outliers removed with RadialMR first).

File name: Supplementary Data 18
Description: Instrument sensitivity analysis removing instruments with outcome p-values <0.05 (outliers detected and removed with RadialMR prior) for the meta-analytic Mendelian randomization (MR) of UK Biobank-measured (circulating, non-fasted) low-density lipoprotein cholesterol (LDL) on healthspan.

File name: Supplementary Data 19
Description: Mendelian Randomization Pleiotropy RESidual Sum and Outlier (MR PRESSO) sensitivity analysis of the meta-analytic MR of UK Biobank-measured (circulating, non-fasted) low-density lipoprotein cholesterol (LDL) on healthspan (outliers removed with RadialMR first).

File name: Supplementary Data 20
Description: Instrument sensitivity analysis removing instruments with outcome p-values <0.05 (outliers detected and removed with RadialMR prior) for the meta-analytic Mendelian randomization (MR) of UK Biobank-measured (circulating, non-fasted) apolipoprotein B (APOB) on Alzheimer's disease.

File name: Supplementary Data 21
Description: Mendelian Randomization Pleiotropy RESidual Sum and Outlier (MR PRESSO) sensitivity analysis of the meta-analytic MR of UK Biobank-measured (circulating, non-fasted) apolipoprotein B (APOB) on Alzheimer's disease (no outliers removed with RadialMR).

File name: Supplementary Data 22
Description: Mendelian Randomization Pleiotropy RESidual Sum and Outlier (MR PRESSO) sensitivity analysis of the meta-analytic MR of UK Biobank-measured (circulating, non-fasted) apolipoprotein B (APOB) on Alzheimer's disease (outliers removed with RadialMR first).

File name: Supplementary Data 23
Description: Instrument sensitivity analysis removing instruments with outcome p-values <0.05 (outliers detected and removed with RadialMR prior) for the meta-analytic Mendelian randomization (MR) of UK Biobank-measured (circulating, non-fasted) low-density lipoprotein cholesterol (LDL) on Alzheimer's disease.

File name: Supplementary Data 24
Description: Mendelian Randomization Pleiotropy RESidual Sum and Outlier (MR PRESSO) sensitivity analysis of the meta-analytic MR of UK Biobank-measured (circulating, non-fasted) low-density lipoprotein cholesterol (LDL) on Alzheimer's disease (outliers removed with RadialMR first).

File name: Supplementary Data 25
Description: MR-PRESSO sensitivity analysis of the meta-analytic MR of UK Biobank-measured (circulating, non-fasted) APOB on Jansen Alzheimer's disease (outliers removed with RadialMR first).

File name: Supplementary Data 26
Description: MRlap sensitivity analysis of the UK Biobank-measured (circulating, non-fasted) apolipoprotein B (APOB) on healthspan.

File name: Supplementary Data 27
Description: Genetic covariance intercept sensitivity analysis of the summary statistics for Jansen et al. (2019) Alzheimer's disease (AD) with Kunkle et al. (2019) AD, UK Biobank (UKBB) apolipoprotein B (APOB), parental lifespan, healthspan, and UKBB low-density lipoprotein cholesterol (LDL).
